# Supplementary material for: Are carbon emissions trading and green financial instruments synergistic? -Comprehensive quantitative research based on content analysis
Source: PLoS One. 2024 Mar 7;19(3):e0298601. doi: 10.1371/journal.pone.0298601 (PMC10919601; doi:10.1371/journal.pone.0298601)
Supplement: S1 Data — (ZIP) [file pone.0298601.s001.zip › Data/地级市Z_F工作报告.zip!/╕╩╦α/╟∞╤⌠╩╨/└┤╫╘╣1⁄2╓┌║┼í╛┬φ┐╦╩2╛▌═°í┐_2018.docx]

各位代表：

　　现在，我代表市人民政府向大会作工作报告，请予审议，并请各位政协委员和列席人员提出意见。

2017年政府工作回顾

　　2017年，在省委、省政府和市委的正确领导下，全市上下深入贯彻习近平新时代中国特色社会主义思想，全面落实习近平总书记视察甘肃重要讲话和“八个着力”重要指示精神，主动适应经济发展新常态，统筹推进稳增长、促改革、调结构、惠民生各项工作，全力推进“三重”、“三一”措施落实,全市经济社会持续稳定健康发展。

　　——经济运行缓中趋好。预计到年底，全市完成生产总值600亿元，增长4%。社会消费品零售总额240亿元，增长7.5%。财政总收入147亿元，增长7.1%。一般公共预算收入45.8亿元，增长8.5%。经济总体上呈现出速度放缓但质量趋好的势头。

　　——脱贫攻坚成效显著。经市级核查验收，全年可脱贫7万人以上，贫困发生率由2016年底的12.2%降至9.2%以下，西峰区、正宁县有望脱贫摘帽。

　　——基础支撑持续强化。实施基础建设项目306个，完成投资153亿元，银西高铁庆阳段、甜永高速公路等一批事关当前和长远发展的重大项目取得突破性进展，交通、水利、城镇等基础设施条件进一步改善。

　　——环境整治力度空前。扎实推进环境突出问题整改，全力打响“蓝天保卫战”，深入开展全域无垃圾专项治理行动，主要污染物排放量呈下降趋势，市区优良天气率达到89%。

　　——人民生活水平稳步提高。全市城镇新增就业3.96万人，登记失业率2.5%。城镇居民人均可支配收入27375元，增长8.2%。农村居民人均可支配收入8116元，增长8.5%。

　　一年来，我们突出抓了以下七个方面的工作：

　　一是全力推进脱贫攻坚。将脱贫攻坚与“三农”工作有机结合起来，落实“五个一批”和“绣花式”扶贫措施，大力实施产业扶贫，推广全膜双垄沟播面积350多万亩，粮食总产量达到130多万吨。新栽苹果11万亩，累计达到177万亩，苹果产量达到90多万吨，预计销售收入近40亿元。肉羊、肉牛、生猪和肉鸡饲养量分别达到672万只、84万头、179万头和3860万只。种植瓜菜150万亩。大力实施基础扶贫，新修农村公路1800多公里，实施生命安全防护工程1000多公里。开工建设易地扶贫搬迁安置点144处，改造危窑（房）11560户，7.6万农村居民住上了安全房屋。12.6万人安全饮水问题得到有效解决。大力实施生态扶贫，为贫困户兑付退耕还林、天然林保护和生态公益林补偿资金5243万元，从贫困人口中聘用护林员2112人、发放补助资金1689万元。大力实施教育扶贫，7.7万贫困家庭学生享受资助政策，农村贫困学生义务教育巩固率明显提高。大力实施兜底扶贫，2.5万名贫困患者享受基本医保、大病救助、医疗救助等特惠政策，农村低保受益面达到13.9%。大力实施金融扶贫，累计发放扶贫再贷款30.5亿元，撬动新增涉农贷款和扶贫贷款65.4亿元。农村产业发展资金专业合作社试点工作扎实推进，累计为14.4万农户发放贷款48.2亿元。便民金融服务点覆盖了95%的行政村，金融服务便利化程度进一步提高。持续加强帮扶工作，全市2万多名干部结对帮扶5万多户贫困户，天津市5区对口帮扶我市5 县和中央、省直单位定点帮扶不断深化，社会多方面力量参与脱贫攻坚的格局已经形成。

　　二是培育壮大优势产业。坚定推进首位产业，加强与国家有关部委和央企的汇报衔接，加快能源开发重点项目建设。原油产量、加工量再创新高，预计分别达到784万吨和355万吨，较上年分别增加20.8万吨、11.6万吨。瑞华天然气深加工、永欣石化氢化石油树脂项目基本建成，庆阳能化集团压缩天然气生产装置建成投产，加工销售天然气364万立方米。核桃峪矿井二期工程即将完成，新庄矿井顺利复工、二期工程启动实施，甜水堡2号矿井取得国家能源局产能置换批复，刘园子矿井接续采煤工作面巷道掘进加快实施。培育壮大中小企业，新建改扩建农产品加工企业20户，落实“助保贷”、“互助贷”等扶持政策，协调向120户企业发放贷款4.3亿元。深入开展“一对一”服务，帮助8户企业恢复生产。大力推广“中盛”、“海升”全产业链发展模式，千万只肉羊产业化基地建设项目列入全国农业领域第一批PPP试点，环县中盛百万只肉羊屠宰加工项目建成投产，宁县海越苹果分拣储藏项目开工建设。大力发展文化旅游产业，庆城县药王洞养生小镇、镇原县北石窟驿、西峰蒲河川区等景区景点建成运营。全市接待游客840万人次，旅游综合收入42亿元，增长34%。加快发展信息产业，华为云计算大数据中心开工建设，与上海联影、软通动力等6户关联企业签订了框架合作协议。一批电商企业蓬勃兴起，市县乡村四级电商服务网络基本形成，省政府在我市召开了全省电子商务进农村综合示范暨电商扶贫经验交流会。全市电商交易额达到26亿元，增长25%。

　　三是加快基础设施建设。落实重大项目市级领导协调抓促责任制，实施500万元以上项目951个。银西高铁庆阳段建设快速推进，庆平铁路完成可研。贯通我市南北的甜水堡至永和高速公路全线开工建设，南梁至太白、打扮梁至庆城、庆阳至平凉3条高速公路PPP项目试验段建设加快推进。华池通用机场土方工程全面完成。五台山、莲花寺水库完成主体工程，小盘河水库完成65%的工程量。马莲河水利枢纽工程水利部正在组织可研评审。海绵城市试点工作有序推进，22个试点项目完成投资4亿元。市区集中供热改造工程基本完成，新增集中供热面积730多万平方米，累计达到1400多万平方米，集中供热覆盖率达到93%。7县县城新增供热面积230多万平方米。北师大庆阳附属学校如期建成并招生。市第二人民医院完成整体搬迁，市妇女儿童医院完成主体工程，市养老综合服务中心一期工程基本建成即将投用。庆阳传媒中心、庆阳大剧院基本完成主体工程。

　　四是大力改善生态环境。全面落实《庆阳市禁牧条例》及退耕还林政策，深入实施“再造一个子午岭”、固沟保塬和资源开发区生态治理工程，完成造林100万亩以上。新建、维修加固淤地坝36座，新修梯田22万亩，治理流域600多平方公里。全面落实抑尘、控煤、治污和重点区域整治等综合措施，组织实施“蓝天保卫战”，全市淘汰10蒸吨及以下燃煤锅炉420台，改造20蒸吨以上燃煤锅炉39台、淘汰停用5台。巴家嘴水库集中式水源地环境治理全面加强，保护区内滩涂地基本退出，一级保护区内耕地退出、居民搬迁工作进展顺利。各级水源地保护区共关闭退出油气水井219口，子午岭自然保护区内128口油气水井全部封堵退出。中央环保督察组交办的126个信访投诉案件全部办结；反馈意见涉及我市的22个问题，整改完成16个，正在整改6个。省环保督察组交办的260个信访投诉案件已办结230件。积极推行“河长制”，境内重要河流沿线乡镇污水直排、河道采砂等问题得到有效遏制。在全域无垃圾专项治理行动中，清理垃圾近13万吨，拆除乱搭乱建近1万处，新建垃圾收集点900多处、转运站11处、污水处理厂11座。建成美丽示范村88个，城乡人居环境显著改善。

　　五是着力增进民生福祉。优先发展教育事业，新建、改扩建行政村幼儿园286所，改造薄弱学校750所，建成“智慧校园”项目7个，培训专业教师8254人，学前教育三年毛入园率达到94%，全市义务教育巩固率达到96%，高中阶段毛入学率达到91%，普通高考本科以上进线率达到59.6%。积极推进分级诊疗，加强全科医生培训，医疗服务水平进一步提升。繁荣发展公共文化事业，全面落实“三馆一站一屋”免费开放，实现了行政村“乡村舞台”全覆盖。全民健身活动深入开展，竞技体育水平稳步提高。加快科技创新应用与成果推广，专利申请突破2000件。健全完善社会保障体系，全民参保计划有序推进，机关事业单位养老保险制度改革启动实施，城乡低保、农村特困人员救助供养补助标准进一步提高。棚户区改造开工建设住房10832套，基本建成10086套，货币化安置率达到89%以上。市区48条主次干道、42条巷道综合治理和20处环卫工人休息室及文明公厕修建等省市列民生实事项目全部落实到位。加强市场价格监管，西峰城区居民用气价格每立方米下调0.8元。全面完成市区公交运营体制改革，投放纯电动公交车60辆，新增公共自行车1000辆，极大地方便了市民出行。实施“宽带乡村”示范工程，实现了村级光纤网络全覆盖。创新社会治理方式，加强信访维稳工作，大力开展矛盾纠纷大排查、大调处活动，全市社会大局和谐稳定。统计、民族宗教、地震、人防、老龄、档案、公积金管理、妇女儿童、残联、红十字会、气象、消防、双拥、国防动员和民兵预备役等方面工作，都有了新的进步和提高。

　　六是持续深化改革开放。积极推进医药卫生体制改革，全面实行公立医院药品采购“两票制”和药品零差率销售，在全国首创组建了市级城乡居民健康保障局，8县（区）机构组建工作也基本完成，实现了“三保合一”和异地就医结算，被国务院医改办确定为公立医院综合改革首批国家级示范城市。创新推动安全生产领域改革发展，成立了全市重大安全协调委员会，统筹协调生产安全、环境安全和食品药品安全工作，形成了“大安全”工作格局。建立重大安全隐患和问题市级领导包抓机制，深入开展安全生产大检查和重点领域专项整治，在全省率先实现农村公路交管全覆盖，并实施了生命安全防护工程，全市生产安全事故起数、死亡人数、受伤人数、直接经济损失“四项指标”全面下降。深入推进“放管服”改革，市本级取消、调整和下放行政审批事项38项，486项审批服务事项集中进驻市政府政务服务大厅。推进“互联网+政务服务”，线上线下联动互补的政务服务平台基本建成。积极推进商事制度改革，“多证合一、一照一码”得到落实。大众创业、万众创新态势良好，新增登记市场主体1.8万户。持续推进农村改革，土地确权通过省上验收，土地流转规模累计达到166万亩。进一步扩大对外开放合作，加强与美国埃德蒙市、日本石川县等城市的教育、文化、经贸交流合作。积极参加“兰洽会”、“津洽会”、“敦煌文博会”和“民企陇上行”等活动，签约实施了一批招商引资项目，北京正大、圣元环保、河北微聚等126户有实力的民营企业落户我市。预计全年实施500万元以上招商引资项目301个，到位资金560亿元。

　　七是不断加强政府建设。全面推进依法行政，严格执行政府工作规则和重大事项决策程序，认真落实公众参与、专家论证、风险评估、合法性审查和集体讨论决定等制度，坚持“三重一大”事项提交市委审定，行政决策的科学化、民主化、法治化水平进一步提高。自觉接受人大依法监督、政协民主监督和社会各界监督，办理人大代表建议115件、政协委员提案315件。加强政府系统廉政建设，着力提升政府效能。深入推进“两学一做”学习教育常态化制度化，扎实开展“明查暗访督查年”活动，行政监察和审计监督进一步加强，正风肃纪更加严明，立案审查政府系统公职人员123人，其中党纪处分100人、政纪处分64人、组织处理22人，约谈问责72人。

　　各位代表，今年我市经济社会发展的成就来之不易，这是省委、省政府和市委正确领导的结果，是市人大、市政协和社会各界鼎力支持的结果，是全市人民同心同德、真抓实干的结果。在此，我谨代表市人民政府，向各位代表和政协委员，向全市各行各业的劳动者，向各民主党派、工商联、无党派人士和人民团体，向驻庆部队和中央、省属驻庆单位，向所有关心支持庆阳发展的人士，表示崇高的敬意和衷心的感谢！

　　在总结成绩的同时，我们也清醒地认识到，全市经济社会发展还面临一些比较突出的困难和问题，政府工作还存在一定差距和不足。一是经济增速放缓，部分指标未能实现预期。受国家产业和财政金融政策调整，以及环保安全政策趋紧、监管趋严影响，一些能源类项目建设受阻、推进缓慢。一些中小企业运营困难，14户规模以上工业企业停产，一批采砂、砖瓦等企业关停整顿。农业因春夏连续干旱、秋季阴雨低温造成较大减产减收。预计规模以上工业增加值完成230亿元，固定资产投资完成394亿元，生产总值完成600亿元，与市四届人大一次会议确定的目标任务有较大差距。二是县域经济实力较弱，脱贫攻坚任务艰巨。县（区）财政普遍困难，财政自给率平均只有11.5%，一些县（区）债务较大，保障发展能力不足。全市深度贫困人口占比大，贫困村集体经济薄弱，贫困群众收入低，脱贫难度大。三是城镇化水平低，基础瓶颈制约尚未消除。全市城镇化率仅为34.9%，比全国、全省分别低22.3和9.7个百分点，城镇功能不完善，辐射带动作用弱。水资源相对短缺，高等级公路里程少，城乡污水、垃圾处理设施滞后，环境保护形势严峻。四是干部能力作风与发展需要不相适应，政府执行力有待进一步提高。政府系统部分工作人员能力不足、动力不足、担当不足，统筹谋划、推动落实、解决问题有差距，“怕”字当头、顾虑重重，等等，致使一些工作落实不到位，效果不理想。

　　这些问题，既是制约全市经济社会发展的瓶颈短板，也是人民群众关心关注的热点难点，更是今后工作的着力点和突破口。我们将坚持问题导向，着力破解难题，以更加坚决的态度、更加有效的措施、更加务实的作风，努力把政府各项工作做实做好，不辜负全市人民的期望和重托。

　　2018年主要工作任务

　　按照中央、省委省政府和市委的决策部署，统筹考虑宏观经济形势和我市发展实际，2018年政府工作的总体要求是：全面贯彻党的十九大精神，以习近平新时代中国特色社会主义思想为指导，坚持稳中求进、质量第一、效益优先，围绕全面建成小康社会，突出抓重点、补短板、强弱项，坚决打好精准脱贫、污染防治、防范风险三大攻坚战，着力在乡村振兴、县域经济、工业强市、新兴产业、基础建设、改革开放、改善民生等重点工作上下功夫、求实效，全力推动经济社会持续健康发展。

　　经济社会发展预期目标是：生产总值增长5%，固定资产投资总额增长8%，社会消费品零售总额增长7.5%，财政总收入增长5%，一般公共预算收入增长7%，城镇居民人均可支配收入增长8%，农村居民人均可支配收入增长9%。各项约束性指标控制在省上下达的计划之内。

　　主要工作任务是：

　　（一）实施乡村振兴战略，壮大县域经济实力，强力攻坚精准脱贫。

　　以实施乡村振兴战略统揽“三农”工作全局，按照产业兴旺、生态宜居、乡风文明、治理有效、生活富裕的总要求，大力发展县域经济，深入实施脱贫攻坚“一号工程”，促进精准施策到村到户，确保全年脱贫8万人以上。

　　全力抓促培育增收产业。坚持以产业发展促进脱贫攻坚，持续不懈地推进草畜、苹果、瓜菜、苗林等特色产业。加快中盛肉羊肉鸡、正大生猪饲养基地建设，全市肉羊、肉牛、生猪、肉鸡饲养量分别达到770万只、89万头、185万头、4000万只。新栽苹果10万亩。种植瓜菜150万亩。发展苗林100万亩。因地制宜发展小杂粮、中药材、黄花菜等传统优势产业，多渠道增加群众收入。

　　聚焦解决深度贫困问题。紧盯全市2个深度贫困县、3个深度贫困乡、230个深度贫困村，积极探索推广肉羊产业扶贫模式，促进贫困户脱贫和村级集体经济发展。上半年在庆城、环县、镇原、华池4县部分乡镇的贫困村先行试点“331+”肉羊产业扶贫模式，随后全面推开。力争通过3年努力，使贫困户依靠肉羊产业实现稳定脱贫。对标对表“两不愁、三保障”目标，着力解决通村（组）道路、饮水、用电、住房等方面的实际困难，确保在有限的时间内解决有限的问题，确保完成深度贫困地区脱贫年度计划。

　　做大做强县域经济。按照市委、市政府提出的“一年初见成效、三年大见成效”的县域经济发展目标，着力推进县域农业产业化、工业集群化、服务业多元化，大力培育一批特色鲜明、辐射范围广、发展潜力大的特色产业集群，夯实县域财源基础，壮大县域经济总量。加大向县（区）下放项目受理、审查、审批等权限的力度，以“扩权强县”改革激发县域经济发展活力。积极争取省上2500亿元专项贷款和产业投资基金的支持，市财政整合安排1亿元发展资金。落实新增建设用地向县域倾斜等优惠政策，优先保证园区建设、重大项目、重大产业和招商引资项目的用地需求，多渠道解决县域经济发展资金难题，支持推动县域经济加快发展。力争到2020年，县（区）经济实力明显增强、质量效益同步提升，西峰区进入全省前10强行列，庆城县、环县、华池县进入全省前20强行列。

　　深化农村综合改革。以农村“三变”改革为牵引，完成农村承包地颁证工作，加快宅基地和集体建设用地使用权确权登记，深入推进农村土地集体所有权、农户承包权、土地经营权分置，健全农村产权交易市场体系，严格落实“一户一宅”农宅管理制度，建立废旧宅基地退出复垦机制，新增耕地指标依规在省内交易，环县、镇原两个深度贫困县土地指标可跨省区交易，收益所得优先用于脱贫攻坚工作。引导土地向经营主体集中，放活土地经营权，鼓励农民自愿扩大土地流转，促进土地入股联营，发展适度规模经营，把有种养意愿的农户培育成种养大户，把种养大户培育成家庭农场，以住房使用权等资源资产入股方式合作发展乡村旅游服务产业，将各类资金集中入股企业或合作社，建立利益联结机制，促进资源变资产、资金变股金、农民变股东，确保群众实现持续稳定收入。大力扶持村级组织领办创办专业合作社、农产品生产加工企业等新型经营主体，将存量农村集体产权作价入股，获得集体收入，壮大村集体经济。加快户籍制度改革，完善推进城乡一体化各项配套措施，逐步破除制约城乡均衡发展的体制机制性障碍，切实提高农村综合改革的系统性、整体性和协调性。

　　完善农村基础设施。改造县乡道路870公里，建设撤并建制村公路630公里，硬化村组道路3500公里。新建、维修农村饮水安全提升工程50处，新建分散供水工程2800处，解决14万人安全饮水问题。改造建档立卡贫困户、低保户、农村分散供养特困人员、贫困残疾人家庭等四类重点对象C级危房5400户以上。深入推进易地扶贫搬迁，搬迁安置群众4900户19600人。改造10千伏输电线路55公里，着力补齐农村基础短板，进一步改善群众生产生活条件。

　　推进农村环境整治。深入开展全域无垃圾专项治理行动，治理农村面源污染，实施垃圾强制分类，下大力气解决“脏乱差”问题。建立农村垃圾“户分类、组收集、村转运、县乡处置”机制，加快乡镇垃圾转运站、填埋场和污水处理站建设。着力推进“厕所革命”，逐步从景区景点扩展到全域、从城市扩展到农村、从数量增加到质量提升，让群众用上卫生的厕所。通过农村环境综合整治，提升全市农村风貌，建设生态宜居的美丽乡村。

　　加强农民教育培训。坚持扶贫与扶志、扶智相结合，有针对性地开展职业技能菜单式培训，提高贫困群众就业创业能力，培养有技术、懂生产、会经营的新型农民。引导激励贫困群众树立主体意识，克服等靠要思想，增强勤劳致富的主动性和自信心。推动乡村移风易俗，破除天价彩礼、婚丧大操大办等陋习。加强农村社会治理，整治聚众赌博行为，打击村霸和宗族恶势力，形成乡风文明新风尚。

　　做细做实帮扶工作。进一步加强与天津市对口帮扶和中省单位定点帮扶，引进一批先进企业，落地实施一批帮扶项目，引导全社会参与扶贫开发，形成脱贫攻坚的强大合力。选派能力强、肯吃苦的优秀干部到村帮扶，加强贫困村“第一书记”和驻村工作人员管理。加强农村基层基础工作，健全乡村治理体系，培养造就一支懂农业、爱农村、爱农民的“三农”工作队伍。加强扶贫资金管理使用，严厉查处贪污、挪用扶贫款项行为，促进阳光扶贫、廉洁扶贫。

　　（二）深入推进工业强市战略，培育壮大产业集群。

　　坚持工业强市不动摇，大中小工业并举，质量效益并重，坚定不移推进能源首位产业开发，不断增强工业经济实力，努力培育优势突出、特色鲜明的工业体系。

　　持续推进油气产业开发。全力支持长庆油田增储上产，支持玉门油田加快环县木西区块开发，支持华北油田加大复产力度，争取全年原油产量达到810万吨以上。支持庆阳石化公司在确保完成大检修的情况下力争加工量达到330万吨以上。力促瑞华天然气深加工、永欣石化氢化石油树脂项目尽快投产。加大境内天然气勘探开发力度，建设环县至宁县长庆桥气源工程主干管道，力争生产销售天然气3亿立方米。锲而不舍地争取实施庆阳石化600万吨炼油升级改造项目。

　　全力抓好煤电项目建设。加强入驻煤电开发企业协调力度，支持华能庆阳煤电公司加快核桃峪矿井建设，确保年内出工程煤50万吨。持续推进新庄矿井建设。支持大唐煤业公司加快刘园子矿井接续采煤工作面巷道掘进，尽快达产达标。加快办理马福川矿井相关手续，争取开工建设。力争毛家川矿井取得“路条”。加快推进甜水堡2号矿井项目核准，力争尽快投产。督促甘肃能源庆阳煤电公司加快推进九龙川矿井前期工作。跟踪推进陇东±800千伏特高压直流外送工程和正宁电厂、环县电厂等重大项目。

　　着力培育新的工业增长点。围绕油煤气装备制造维修、绿色农产品精深加工、民俗文化和新能源、新材料等领域，支持兴办一批工业企业，形成多点支撑的工业发展格局。加快庆阳华为云计算大数据中心建设，带动更多关联企业落户我市，加快建设信息产业孵化园，努力形成区域大数据处理中心和全省信息消费中心。加快肉鸡、肉羊、生猪屠宰加工、秸秆饲料化利用和苹果分拣储藏等产业化项目建设。全面落实扶持政策措施，理直气壮地支持工业企业发展，引导中小企业改进生产技术，建立现代企业管理制度，提高生产经营效益。加强政银、银企合作，扩大“助保贷”、“互助贷”规模，帮助中小微企业解决生产经营中的实际困难，促使正常生产运行。进一步理顺工业园区管理体制，加快园区“七通一平”基础设施建设，吸引更多企业入驻园区、投资发展。

　　（三）抓项目扩投资，大力改善基础设施条件。

　　牢固树立发展抓项目的理念，围绕政策导向抓谋划争取，紧盯形象进度抓建设管理，扩大有效投资，以项目工作的突破拉动经济稳定增长。

　　推进重大基础建设。交通方面，全力支持银西高铁庆阳段、甜水堡至永和高速公路加快建设进度，抓紧推动南梁至太白、打扮梁至庆城高速公路和二十里沟口至车路崾岘高等级公路全线开工建设，争取庆阳至平凉高速公路开工建设，加快7条355公里省道改建项目建设。积极推进庆平铁路、庆阳综合交通枢纽项目前期工作，建成华池通用机场，争取庆阳至天津、庆阳至西南航线开通运营。水利方面，建成五台山、莲花寺水库并试运行，完成小盘河水库主体工程。力争马莲河水利枢纽工程批复立项，做好葫芦河引水、扬黄人饮向市区供水项目前期工作。开工建设董志塬水厂、市区东部污泥处理厂和南部工业园区污水处理再生利用工程，建成市区东部污水处理厂。电力方面，加快推进庆中、洪德两个330千伏输变电工程建设，新建110千伏输变电工程3个、35千伏输变电工程5个。城镇方面，加快推进城市总体规划修编暨“多规合一”规划、控制性详细规划编制，切实加强城市规划工作，依法强化规划的控制作用。统筹抓好市区、县城和小城镇建设，创建特色小镇10个。投资100亿元，实施城建项目280项。扎实推进海绵城市试点工作，加快市区北部雨水收集利用、南部生态绿地、公共停车场、市政道路等基础设施建设，进一步完善县城供排水、集中供热、供气等配套设施。采取货币化安置、异地回购等方式，实施35个片区5300多户棚户区改造工程。全市城镇化率提高1.5个百分点。

　　加强项目谋划争取。紧盯国家推进“一带一路”建设、供给侧结构性改革、支持深度贫困地区脱贫攻坚等政策导向，加大项目谋划力度，加强与国家有关部委、省直相关部门的衔接争取，力争更多项目进入国家和省上计划“盘子”。加大项目前期论证投入，动态完善重大项目库，谋划、储备、培育、实施一批能够支撑带动全市经济社会发展的大项目好项目。

　　加大项目招引力度。认真贯彻省政府关于加强招商引资促进外资增长的24条措施，进一步放宽准入限制，落实税费优惠政策，加大金融扶持力度，完善招商引资激励机制。紧盯与我市产业关联度强的核心优势企业，把招商引资的触角延伸到技术先进、民资富集和产业集聚的地区，开展上门招商、精准招商、全产业链招商，吸引有实力、有潜力、有带动性的大型企业落户我市。全力推动西峰垃圾焚烧发电、长庆桥微聚科技产业园、驿马中盛产业园等项目顺利实施，确保招商引资项目到位资金增长10%以上。

　　（四）促进多元融合，推动服务业加快发展。

　　把发展壮大第三产业作为新的经济增长点，加快培育新业态，促进产业多元融合发展，有效提升经济发展质量和效益。

　　做大做强文化旅游业。按照“全域布局、互补发展、差异竞争”的思路，突出县域特色，实行错位布局，全市统一谋划，串点成线、连线成面，推动旅游产业由景区旅游向全域旅游转变，由单一业态向综合产业转变。积极推进“旅游+”跨界融合，深度开发旅游产品，完善配套功能，推广采摘体验基地、田园综合体等发展模式，培育全要素旅游消费热点，着力构建观光、休闲、度假、康养全面发展的旅游产业体系。加快庆阳旅游品牌建设，统筹规划旅游线路，重点打造以南梁大景区为中心的红色旅游线，加快建设蒲河川、湘乐川和子午岭林缘区三条文化旅游风情线，实施好西峰月亮湾香草园、镇原屯字药王谷等一批重点项目，建设西峰、庆城两个游客集散中心，努力打造知名度高、影响力强的全域旅游目的地。

　　推进电子商务和现代物流业发展。建成镇原中盛、西峰天禄冷链物流中心，实施电商进农村综合示范项目，推广网上交易、电子支付等方式，支持物流快递业发展，完善电商供应链条和服务体系，扩大电商交易规模，力争电子商务交易额增长20%以上。

　　大力发展养老服务业。以健康老龄化为目标，科学统筹医疗卫生和养老服务资源，健全制度，创新模式，大力推进医养结合项目，建立健全互联网+医疗+养老综合服务平台，促进老年护理、医养结合等新产业、新业态发展，更好地满足老年人的医养服务需求。

　　改造提升传统服务业。大力支持交通运输、商贸流通、住宿餐饮等传统业态改造升级，引导相关行业主体更新产品、提升质量、优化服务，推动生产型服务业向专业化和价值链高端延伸，促进生活型服务业向精细化和高品质方向转变。进一步健全完善市场流通服务体系，引导扩大教育、文化、体育和汽车、住房等消费，提高消费对经济增长的贡献率。

　　强化金融保障支撑。完善融资体系，支持中盛农牧、中庆农产品等有条件的企业上市或在“新三板”、区域性股权交易中心挂牌融资。大力发展普惠金融，鼓励金融机构围绕支持小微企业、精准扶贫等领域，量身订制金融产品，扩大信贷投放规模，提高金融服务经济发展的效益和水平。加快保险市场建设，开展保险扶贫试点，实现保险网点行政村全覆盖，提升保险业保障服务能力。优化金融生态环境，整顿规范民间投融资市场，严厉打击非法集资、金融诈骗、恶意逃废银行债务等违法犯罪活动，有效防范金融风险。

　　（五）抓治理强监管，确保生态环境持续好转。

　　秉持“绿水青山就是金山银山”的理念，统筹推进生态环境保护和重点领域治理，着力提升环境承载能力，改善生态环境质量，努力建设山清水秀、天蓝地绿、空气清新、环境整洁的美丽庆阳。

　　抓好生态保护与建设。深入实施“再造一个子午岭”工程，扎实推进“三北”防护林、退耕还林、天然林保护等重点生态工程，持续做好荒山、道路、村镇、城区和矿区绿化，完成造林绿化100万亩以上。深入实施固沟保塬工程，严格保护耕地，建设高标准基本农田12万亩以上。强化水土流失治理，新修梯田15万亩以上，新建和加固病险淤地坝40座，治理水土流失600平方公里。

　　着力解决突出环境问题。全面落实“大气十条”、“水十条”、“土十条”，继续抓好中央、省上环保督察反馈问题整改，坚决打好“蓝天保卫战”，严控燃煤、扬尘污染，推进污染源达标排放。严格落实“河长制”，坚决整治沿河排污口，规范河道采砂。加强子午岭自然保护区、饮用水源地保护，完成巴家嘴水库水源地一级保护区耕地退出和居民搬迁工作。扎实开展土壤污染详查，推进油田废弃场地等重点区域土壤污染防治与修复工作。

　　加强环境执法监管。全面落实《庆阳市禁牧条例》，出台具体实施办法，巩固造林绿化成果。完善网格化环境监管体系，深入开展环境隐患排查整治，严厉打击环境违法行为。对破坏生态环境的典型案件，公开曝光，严肃追责和查处。

　　（六）促创新增活力，纵深推进改革开放。

　　贯彻落实中央和省委省政府、市委关于全面深化改革的各项决策部署，向改革要动力，向开放要活力，努力为全市发展营造良好的环境。

　　统筹推进重点领域改革。深化医药卫生体制改革，按照“改革为民、过程便民、结果惠民”的导向和“政府能负担、医院可持续、群众得实惠”的思路，持续纵深推进城乡医改工作。尽快建立健全市、县（区）城乡居民健康保障局运行机制，加快“三保合一”步伐，建立完善现代医院管理制度，不断提高医疗水平，使优质医疗服务更多地惠及广大人民群众。深化“放管服”改革，及时承接落实国务院、省政府取消下放的行政审批事项；加快推进政务信息系统共享，推动市县乡村“互联网+政务服务”平台一体化，实现政务数据资源互通共享。加快市县两级行政许可标准化建设和“减证便民”事项清理工作，促进行政审批规范化运行，切实提高行政效能和服务水平。深化财政制度改革，规范政府举债行为，强化预算约束，优化支出结构，全面推行国库集中支付电子化改革。严格执行公务卡强制结算目录，扎实推进全口径预算管理，强化财政绩效管理，加强财政投资绩效评价，切实提高财政资金使用效益。深化城市综合执法体制改革，完善城市管理标准规范，推动城市管理重心下移、职责下沉，强化城管执法人员管理，提高城市管理水平。深化环保管理体制改革，配合做好环保机构监测监察执法垂直管理制度改革相关工作。深化国资国企改革，完善法人治理结构，建立现代产权制度和现代企业制度，推进混合所有制改革。推进行政事业单位体制机制创新，鼓励引导各行业部门创新理念、改进方式、破解难题、促进发展，建立健全正向激励、容错纠错机制，充分调动各级干部干事创业的积极性，推动形成工作创一流、争上游的浓厚氛围。

　　持续扩大对外开放。依托“兰洽会”、“敦煌文博会”等展会平台，大力宣传推介我市各类特色产品，建立由龙头企业牵头的庆阳农特产品销售渠道，扩大产品影响力和市场竞争力。进一步加强与“一带一路”沿线国家和地区、东部沿海发达城市、西咸经济区的经贸往来和文化交流，扩大多层次、全方位的对内对外开放合作，实现区域、企业、产业融合发展。

　　加快非公经济发展。牢固树立“产业第一、企业为大”的理念，理解、尊重、包容、支持企业家发展，真心实意保护企业家合法权益，建立完善政企沟通会商机制，积极构建“亲”、“清”新型政商关系，着力提升政府服务质量，改善政府服务环境。兑现落实支持非公经济发展的政策承诺，推行审批环节并联办理，实行重大项目一事一议，强化土地供应、资金支持等要素保障，落实同等待遇，激发民间投资活力，扭转民间投资下滑的不利局面。

　　（七）惠民生保稳定，着力构建和谐社会。

　　坚持把保障和改善民生作为工作的出发点和落脚点，围绕人民群众的现实需要，统筹推进社会事业，扎实办理民生实事，让改革发展成果更公平更充分地惠及全体人民。

　　加快发展社会事业。优先发展教育，推进教育体制机制改革，合理布局教育资源，大力发展学前教育、特殊教育、职业教育和民办教育，提高义务教育质量，加快普及高中阶段教育。全面加强教师队伍建设，解决好教师队伍结构性短缺突出、整体素质需要进一步提升的问题，努力提高师德素养和教育教学质量。实施365所农村义务教育薄弱学校改造和市实验小学迁建、庆阳一中校舍改扩建等项目，着力解决中小学生课外负担重、“择校热”、“大班额”和适龄儿童入园难、入学难的问题。加快庆阳职业技术学院校舍建设，大力培养职业技术人才，确保顺利通过教育部合格评估验收。支持陇东学院建设，增强服务地方发展的能力。推动科技创新，加快国家农业科技示范园区、科技创新孵化中心等平台建设，加强与科研院所、高等院校的合作，加快产品创新、品牌创新、技术创新、产业组织创新，促进科技创新成果向现实生产力转化。繁荣发展文化体育事业，全面实施文艺繁荣“十大工程”，加强文化遗产保护传承，加快庆阳传媒中心、庆阳大剧院建设，推进“两馆一院”前期工作。深入开展“千台大戏送农村”和“文化下乡”活动，加大政府购买公共文化服务力度，不断丰富群众文化体育生活。抓紧备战省第十四届运动会。推进健康庆阳建设，大力实施全民健康计划，扩大家庭医生签约服务覆盖面，加强医务人员专业技术培训和医德医风建设，鼓励引导优质医疗资源向基层下沉，努力提升农村医疗服务水平。加强人口与生育统筹服务管理工作。加快推进市人民医院扩容提能项目前期工作，力争市妇女儿童医院建成投用。

　　做好就业和社会保障工作。强化职业技能培训，鼓励创业带动就业，积极扶持引导高校毕业生到基层、到非公企业就业，确保城镇登记失业率控制在4%以内。积极推进全民参保计划，全面实施机关事业单位基本养老保险制度改革。认真做好城乡低保提标、失地农民养老保险、社会救助和防灾减灾工作，大力发展民政福利和慈善事业，着力维护残疾人、老年人、妇女儿童的合法权益。进一步完善住房供应体系，积极培育和发展住房租赁市场，建立租购并举的住房制度。加快建设城乡统筹、权责清晰、保障适度的多层次社会保障体系。

　　坚持安全发展不动摇。落细落实安全生产领域改革发展任务，突出抓好安全生产标准化、达标常态化和应急救援、职业卫生健康监管等工作。持续强化道路交通、油气管道、危险化学品、消防、煤矿和非煤矿山、建筑工地和人员密集场所等重点领域安全监管。持续实施农村公路生命安全防护工程，依照法定程序做好《庆阳市农村公路超限运输管理条例》出台的相关工作，切实保障人民群众生命财产安全。强化食品药品和产品质量安全监管，确保群众饮食用药等方面的安全。深入开展平安校园创建活动，加强校园周边环境综合治理。

　　进一步完善社会治理体系。扎实推进全国文明城市创建活动，培育和践行社会主义核心价值观，提升市民文明素质。坚持依法治市，深入实施“七五”普法和法律“九进”活动，提高全民法律素质，健全公共法律服务体系，落实法律援助制度。完善城乡社区服务体系，加强居民小区服务管理，实现政府治理和社会调解、居民自治良性互动。加强信用体系建设，建立企业和个人征信数据库，打造“诚信庆阳”。坚持领导干部接访下访制度，畅通信访渠道，有效化解信访矛盾，解决信访积案。完善信息化、立体化社会治安防控、公共安全防控体系，强化网络舆情监测和应急处突，依法打击黄赌毒、盗抢骗等违法犯罪行为，加强油区社会治安综合治理，形成共建共治共享的社会治理格局，维护社会和谐稳定。

　　扎实办好群众关切的具体事项。在全面落实各项惠民政策的基础上，集中有限财力办好8件实事。（1）继续救助城乡困难家庭高考入学学生。（2）继续做好全市医护人员业务提升培训。（3）采取以奖代补方式，支持所有行政村开展全域无垃圾工作。（4）对市区破旧背街小巷进行综合治理。（5）在市区新建3座人行天桥。（6）对市区老旧供水管网进行提质改造。（7）新建改造全市重点旅游景区和市区公厕。（8）在市区新建5处停车场。

　　 加强政府自身建设

　　党的十九大报告提出：要“转变政府职能，深化简政放权，创新监管方式，增强政府公信力和执行力，建设人民满意的服务型政府”。新时代新形势对政府工作提出了新的更高的要求。市政府将认真贯彻落实党的十九大精神，按照为民、务实、清廉的要求，切实加强自身建设，全力推动发展，持续改善民生，努力做到为人民服务、对人民负责、受人民监督、让人民满意。

　　一要旗帜鲜明讲政治。坚定理想信念、保持战略定力，提高政治站位、增强政治定力，树牢“四个意识”，坚决维护党中央权威和集中统一领导，始终在思想上、政治上、行动上同以习近平同志为核心的党中央保持高度一致。严守政治纪律和政治规矩，认真落实中央八项规定实施细则以及省市委实施意见，坚决贯彻中央、省委省政府和市委各项决策部署，确保政令畅通、令行禁止。

　　二要勤政为民破难题。坚持以人民为中心的发展思想，把人民利益摆在至高无上的地位，积极回应社会和公众诉求，关心群众疾苦，倾听群众呼声，了解群众意愿，在幼有所育、学有所教、劳有所得、病有所医、老有所养、住有所居、弱有所扶上不断取得新进展。着眼解决群众最关心、最直接、最现实的利益问题，强化公共服务，补齐民生短板，促进社会公平正义，使人民群众的获得感、幸福感、安全感更加充实、更有保障、更可持续。

　　三要依法行政促公开。坚持“法无授权不可为、法定职责必须为”，依法全面履行政府职能，完善依法行政制度体系，严格规范公正文明执法，推进行政决策科学化、民主化、法治化。自觉接受人大依法监督和政协民主监督，认真办理人大代表建议、政协委员提案，主动听取各民主党派、人民团体和社会各界的意见建议，主动接受社会舆论监督。进一步强化审计监督，切实增强对行政权力的监督制约。规范和扩大政务公开，努力提高政府工作的透明度。

　　四要提升素质强本领。对照习近平总书记提出“增强八个方面本领”的要求，深入开展“不忘初心、牢记使命”主题教育，巩固深化“两学一做”学习教育成果。加强理论学习，用习近平新时代中国特色社会主义思想武装头脑、指导实践。加强政策学习，增强研究政策和转化运用政策的能力。加强业务学习，争做行家里手，提高工作专业化水平。坚持理论联系实际，全面提升谋划推动发展、破解疑难问题、应对风险挑战、驾驭复杂局面的能力和水平。

　　五要改进作风抓落实。始终把事业放在心上、把责任扛在肩上、把工作抓在手上，把谋事、干事、成事统一起来，大力弘扬以实为本、以真为要、以干为先的作风，以钉钉子的精神抓落实，做到说了就办、定了就干，一项一项破解难题，一件一件兴办实事。加大明查暗访力度，强化问责追责，确保各项工作部署落实到位，各类督察、巡查反馈问题整改到位。坚决治庸、治懒、治散、治慢，着力解决不担责怕担责、不作为慢作为的问题，努力提高工作执行力。

　　六要清正廉洁树形象。全面贯彻中央、省委省政府和市委关于加强纪律作风建设各项规定，自觉落实全面从严治党主体责任，认真履行“一岗双责”，积极支持监察体制改革，强化不敢腐的震慑，扎牢不能腐的笼子，增强不想腐的自觉。坚持廉洁从政、从严治政，严格用制度管人、管权、管事，切实加强工程建设、政府采购、土地出让等重点领域专项治理，铁面执纪、严惩贪腐。驰而不息反对“四风”，倡导勤俭节约、减少开支，反对大手大脚、铺张浪费，上级机关为下级机关带好头、作表率，扬正气、树形象，促进各级政府工作人员以俭为荣、以奢为耻，努力建设廉洁型、节约型机关，营造风清气正的良好政治生态。

　　各位代表，奋斗夯实基础，实干改变面貌。让我们以习近平新时代中国特色社会主义思想为指导，在省委、省政府和市委的坚强领导下，在市人大、市政协的监督支持下，不忘初心、牢记使命，锐意进取、埋头苦干，扎扎实实做好各项工作，为全面建成小康社会、建设幸福美好新庆阳而努力奋斗！
